# Stability over Time of the Sperm Motility Biomarker proAKAP4 in Repeated Dog Ejaculates

**DOI:** 10.3390/ani15081160

**Published:** 2025-04-17

**Authors:** Giulia Siena, Alain Fontbonne, Barbara Contiero, Cindy Maenhoudt, Guillaume Robiteau, Sarah Slimani, Nicolas Sergeant, Laurent Tiret, Chiara Milani

**Affiliations:** 1 Department of Animal Medicine, Production and Health, University of Padova, 35020 Legnaro, PD, Italy; giulia.siena@phd.unipd.it (G.S.);; 2 Centre d’Étude en Reproduction des Carnivores, Ecole Nationale Vétérinaire d’Alfort, 7 Avenue du Général de Gaulle, F-94700 Maisons-Alfort, France; alain.fontbonne@vet-alfort.fr (A.F.);; 3 Université Paris-Est Créteil, Inserm, U955 Institut Mondor de Recherche Biomédicale, Relaix Team, École Nationale Vétérinaire d’Alfort, F-94700 Maisons-Alfort, France; laurent.tiret@vet-alfort.fr; 4 SPQI SAS, F-59000 Lille, France; nsergeant@4biodx.com; 5 Univ. Lille, Inserm UMRS1172, CHRU of Lille, F-59045 Lille, France

**Keywords:** dog, spermatozoa, semen analysis, motility biomarkers, proAKAP4

## Abstract

ProAKAP4 is a sperm structural protein involved in motility, capacitation, and fertility. Previous studies suggested it as a suitable marker for canine sperm evaluation. Our study aimed to assess proAKAP4 concentration variations among different ejaculates collected from the same dogs. Fourteen male dogs from 12 different breeds, 1–14 years old and 6.9–95 kg bodyweight, underwent semen collection at least twice (1–4 times) during the same or different days. Sperm concentration, morphology, proAKAP4 concentration, total (TM%), and progressive motility (PM%) were considered. ProAKAP4 values were divided into four classes: ≤15, 15–40, 40–60, and >60 ng/10 M spermatozoa. The time interval between two semen collections was classified as: first collection (T0), semen collection performed 2–3 h after the first one (T0.5) and after ≥1 day (1–36 days, T1). Thirty-three ejaculates were collected. A correlation was found between proAKAP4 classes and TM% (r = 0.40, *p* = 0.049), whereas no correlation was found neither between proAKAP4 and sperm morphology nor for proAKAP4 classes between collection times (T0 vs. T0.5, *p* = 0.655; T0 vs. T1, *p* = 0.564). Our results confirm the correlation between proAKAP4 and TM% in dog semen. ProAKAP4 concentration appears stable in different ejaculates collected from the same dogs. Further studies are needed on the relationship between proAKAP4 and other canine semen parameters and fertility outcomes.

## Simple Summary

In clinical practice, it is common to collect semen samples from the same dog on the same or on consecutive days, especially for cryopreservation or artificial insemination. In such cases, repeating the semen analysis is necessary for each ejaculate. In order to reduce the human and economic resources involved, new studies are needed on semen quality and variations in markers over time. ProAKAP4 is an essential component of spermatozoa tail architecture, involved in sperm motility and a marker of sperm quality. The present study evaluates its variation over time in subsequent semen samples collected from the same dogs. Fourteen male dogs (12 different breeds, 1–14 years old and 6.9–95 kg bodyweight) underwent semen collection at various time points: first semen sample collection, 2–3 h, and/or 1–30 days after the first semen collection. In our study, proAKAP4 concentration does not seem to vary in ejaculates collected from the same dog at different time points, and it was significantly correlated with total motility. We confirm that proAKAP4 is a relevant, specific, and stable biomarker of total sperm motility. This result prompts further multicentric studies on a higher number of dogs and fertility outcomes before it can be confidently translated into the clinic.

## 1. Introduction

Semen evaluation is a complementary exam used in clinical practice. It is one of the most basic and decisive exams used to assess the fertility of a stud dog. In most cases, veterinarians and breeders request this analysis for a preliminary breeding soundness evaluation of the patient to have a picture of the semen characteristics before performing artificial inseminations, semen chilling, or semen freezing [1]. This exam may also be required in cases of subfertility, such as a lack of pregnancy onset in multiple females after mating or artificial insemination [2]. Semen evaluation consists of the analysis of specific characteristics of both seminal plasma and spermatozoa, including the calculation of the sperm concentration, total number of spermatozoa in the collected sample, total and progressive motility, as well as morphological evaluation performed either manually or using a computer-assisted semen analysis, also referred to as CASA [1,2,3,4].

Despite the unquestionable advantage of being performed easily in most specialized reproduction clinics, semen analysis has a few limitations. One of these is that the evaluated semen parameters reflect the previous two months of spermatogenesis. Moreover, it provides no information about motility lifespan. Another limit could be the variability of sperm production, sperm release, and the reduction in or expiration of the extragonadal reserves after too frequently repeated semen collections [5,6,7]. For this reason, the single analysis should be repeated for each semen sample, also when the same dog is collected within the same day. Even if all the considered parameters are in the range of normality and semen is repeatedly used in different females, sometimes a semen sample might not lead to pregnancy. This may be due to some subtle alterations in spermatozoa that cannot be detected with a standard semen analysis. Therefore, an in-depth evaluation of the different specialized cell compartments or molecular markers of sperm is needed to improve both semen quality assessments and, consequently, pregnancy success rates [4,7,8]. For these reasons, new parameters and their combinations are still under study to reach a high-level fertility prediction for fresh and frozen–thawed semen [7,9,10].

In the last decades, the advancement in “-omics” analytical techniques (e.g., genomic, proteomic, etc.) led to the identification of a vast number of molecular markers related to male reproductive functions. Extensive knowledge of the role of many proteins in spermatozoa and seminal plasma still needs to be disclosed. However, some correlations between protein expression and infertility events have been recently described [8,11,12,13,14,15]. Specifically, several proteins have been identified in sperm cells, among which is the A-kinase anchor protein 4 (AKAP4).

AKAP4 has been identified as the most abundant flagellum structural protein localized in the fibrous sheath of the principal piece [5,6]. In motile and alive spermatozoa, the active AKAP4 form is converted from a proAKAP4 precursor polypeptide, translated from a single X-linked *AKAP4* gene. ProAKAP4 is translated during spermiogenesis, generating a protein stock progressively converted into AKAP4 along the spermatozoa life cycle. AKAP4 belongs to the protein kinase A anchoring gene family. They dock the A-kinase in discrete sub-cellular compartments to coordinate the core transduction AMPc-dependent signals regulating flagellum helicoidal motion. AKAP4 also impacts glycolytic energy balance, supporting motility, hypermotility, and spermatozoa capacitation [5,6,16,17,18,19]. Normal ejaculate volume and spermatozoa are produced in murine animals lacking AKAP4 expression. *AKAP4* knocked-out animals are reported to show sperm tail alterations and modified tail thickness, resulting in immotile spermatozoa and then in infertile animals [20,21,22]. The concentration of proAKAP4 in the semen, therefore, reflects the long-lasting motility of spermatozoa, such as the ability to be active and functional in time up to reaching the site of fertilization. Thus, proAKAP4 conversion to AKAP4 is a physiological process that maintains the molecular mechanism and transduction signal that coordinates sperm motility. Moreover, the higher proAKAP4 stock, the better spermatozoa functionality is maintained over the spermatozoa life cycle. Therefore, any alteration in proAKAP4 concentration and metabolism may affect spermatozoa’s flagella motility, capacitation, and fertility. ProAKAP4 defective expression can result from a spermatogenesis dysfunction, reducing the starting pool of proAKAP4 protein. A defective conversion of proAKAP4 to AKAP4 or regulated expression during development and postnatally may coincide with animal fertility, as suggested [23]. In Camelidae, higher levels of proAKAP4 coincide with the reproduction season [24]. ProAKAP4 and AKAP4 are also sensitive to environmental stressors and degraded under oxidative stress and cryopreservation [25,26]. Then, proAKAP4 and indirectly AKAP4 spermatozoa expression can be impaired by many parameters, and the average concentration in the ejaculate is suggested to provide a sperm quality parameter and potentially predict fertility rates in mammals [6,27].

ProAKAP4 was studied as a clinical biomarker of semen quality in clinical settings in different species [7,19,20,21,28,29,30,31,32,33]. A positive correlation between proAKAP4 values and total and progressive motility was found in humans, bulls, pigs, horses, and camels [19,24,27,31,32,33,34,35,36]. In bulls, total and progressive motility seems to be affected by proAKAP4 concentration, whereas the influence of proAKAP4 values on velocity parameters (like VAP, VSL, and VCL) is still controversial [31,35]. Recent studies reported proAKAP4 as a good indicator of sperm motility, viability, and fertility rates in different mammals [19,20,21,28,32,33]. AKAP4 was involved in spermatozoa morphology and several sperm functions, including motility, hypermotility, and capacitation [5,6,16,17,18,19]. In bulls, proAKAP4 concentration in post-thawed semen is reported to be correlated to non-return to heat, indicating this parameter as a potential predictive marker of bull fertility [27]. Moreover, a better fertility rate was found in bulls showing a high concentration of proAKAP4 compared to those showing a lower concentration [28]. In humans, low values of AKAP4 are reported to be involved in male infertility, being related to sperm capacitation and sperm-egg interaction. ProAKAP4 is also reported as an indicator of DNA fragmentation, being higher in semen samples with lower DNA fragmentation [34], and AKAP4 is lower in semen ejaculates showing high radical oxygen species concentration and low motility [37].

In dogs, it was found in the sperm-rich fraction, whereas it was not found in the urethral and prostatic fraction or seminal plasma, like in other species [5,6,17,27]. In the canine species, a positive correlation between proAKAP4 concentration and TM%, PM%, and velocity parameters was reported [36]. Previous studies suggested proAKAP4 as a good marker to be considered while assessing semen quality in fresh and frozen/thawed samples [29,36,38,39]. However, some information is lacking regarding the potential variation of its concentration in repeated samples collected from the same stud dog.

In clinical practice, it is common to collect semen samples from the same dog on the same day or on different days, especially for chilled semen preparation and shipping, cryopreservation, or artificial insemination. In these cases, it is necessary to repeat semen analysis for each ejaculate. We hypothesize that proAKAP4 is a stable parameter that should not change significantly during the same spermatogenetic cycle. However, no data were collected when close semen collections were performed. Therefore, our study aimed to evaluate the variation in proAKAP4 concentration among different ejaculates collected from the same dogs, either on the same day or on different days of semen collection, and to verify the correlation between proAKAP4 concentration and seminal quality parameters, such as motility percentages and morphology abnormalities.

## 2. Materials and Methods

### 2.1. Animals

Fourteen male dogs, from 1 to 14 years of age and 6.9 to 95 kg of body weight, presented to the Centre d’Etude en Reproduction des Carnivores (CERCA) of the Ecole nationale vétérinaire d’Alfort (EnvA) were included in this study. They belonged to twelve different breeds (one Great Dane, one Staffordshire Bull Terrier, one Mastiff, one Belgian Shepherd Malinois, one Golden Retriever, one Pug, one small Munsterlander, one American Staffordshire Terrier, one Border Collie, one Welsh Corgi Pembroke, two Jack Russell, and two Newfoundland). They were collected from January to March 2022 when their owners and breeders asked for either semen evaluation, artificial insemination, or semen freezing and storage in our center. All procedures and parameters assessed in our study were carried out as a part of routine veterinary activities performed for semen analysis, freezing, and artificial insemination with fresh semen requested by the owners of the included studs. Animal manipulations were performed following the European Convention for the Protection of Vertebrate Animals and used for Experimental and Scientific Purposes (Official Journal of the European Union L276/33, 2010). The ethical approval number was 93/2024; moreover, written consent was obtained by the owners of the included dogs. Anamnesis was recorded on the collection day, and a clinical examination was performed. Only clinically healthy stud dogs subjected to at least two semen collections within two months were included. The exclusion criteria were the presence of clinically evident pathologies related to the genital tract (e.g., cryptorchidism) and the administration of drugs, such as corticosteroids or sex hormones, during the six months before semen collection.

### 2.2. Experimental Design

For each dog, a minimum of two and a maximum of four semen collections were performed. The first semen collection was performed at time 0 (T0). Depending on the time interval between the first and the second semen collection, they were classified as time 0.5 (T0.5) when it was performed on the same day or time 1 (T1) when it was performed at least one day (24 h) after the first semen collection. T0.5 semen collection was performed with a minimum of 2 h and a maximum of 3 h after the first semen collection. T1 was performed between one and 36 days after T0. Semen fractions were collected separately using sterile multi-use artificial silicone vaginas connected to a 15 mL sterile polypropylene conical centrifuge Falcon^®^ tube (Corning Incorporated Life Sciences, Tewksbury, MA, USA). All materials were sterilized and prewarmed before use at 37 °C.

#### 2.2.1. Semen Analysis

Each ejaculate was analyzed considering concentration, motility, and morphology parameters. The concentration of the 2nd fraction (sperm-rich fraction) was counted in a 20 µL sample using a commercial photometer calibrated for the canine species (SpermaCue, Minitube, Tiefenbach, Germany). For spermatozoa morphology analytics, 100 spermatozoa were examined after Spermac^®^ staining (Minitube, Tiefenbach, Germany) using an optical microscope at 100× (Olympus BX40 microscope, Olympus, Tokyo, Japan). Sperm abnormalities were classified as a percentage of cytoplasmic droplets, detached heads, and acrosomal, head, intermediate piece, and tail abnormalities [1,2,3]. Total motility (TM%) and progressive motility (PM%) were measured using 20 µm Leja slides in a Computer-Assisted Sperm Analyser (CASA, Ceros II, Hamilton Thorne Inc., Beverly, MA, USA).

#### 2.2.2. ProAKAP4 Analysis

Aliquots of the second fraction were then stored at −80 °C until the proAKAP4 assay was performed on the thawed spermatic fraction using a commercial ELISA sandwich kit (Dog 4MID^®^ Kit, 4BioDx, Lille, France). The proAKAP4 immunoassay is a sandwich-based ELISA with a capturing proAKAP4 antibody and a second AKAP4 antibody coupled to horseradish peroxidase as the detection antibody. Spermatozoa were lysed with a dog semen lysis buffer provided with the Dog 4MID^®^ Kit (4BioDx, Lille, France) according to the manufacturer’s instructions. The resulting lysate was further diluted before loading 150 µL in each well of the ELISA-coated plate. The plate was incubated overnight before adding the detection antibody, followed by adding the chromogen (TMB), according to the manufacturer’s instructions. A standard curve was obtained using proAKAP4 standard ranged with linear concentrations from 4.6 to 150 ng/mL of proAKAP4. The proAKAP4 concentration in ng/10 million spermatozoa was then calculated using a computer-assisted calculation sheet provided by the manufacturer (SPQI, Lille, France).

### 2.3. Statistical Analysis

Statistical analysis was performed using SAS 9.4 (SAS Institute Inc., Cary, NC, USA). ProAKAP4 normality was assessed using a Shapiro–Wilk test. Therefore, based on the relationship between proAKAP4 concentration and semen quality reported by Carracedo et al., 2022 [6], and by the 4BioDx commercial information [40], in our study, proAKAP4 values were divided into four intervals (class 1: ≤15, class 2: 15–40, class 3: 40.01–60, and class 4: >60 ng/10 M spermatozoa), with the highest TM% values measured in semen samples included in interval 4 and the lowest TM% values included in interval 1 [6].

The correlation between proAKAP4 classes and morphology (% of normal spermatozoa, abnormal head, acrosomal abnormalities, abnormal tails, intermediate piece defects, detached heads, and presence of cytoplasmatic droplets), TM% and PM% was assessed using the Spearman rank correlation (r).

ProAKAP4 results, reported as classes, were subjected to a non-parametric paired Wilcoxon test to verify statistical differences among collection times (T0 vs. T0.5 and T0 vs. T1). The Wilcoxon test was also performed to confirm the statistical difference between total motility (TM%), progressive motility (PM%), and concentration of spermatozoa evaluated at T0 and T0.5 and T0 and T1.

Correlations among TM%, PM%, proAKAP4 classes, and semen concentration in ejaculates collected twice during the same day (T0 and T0.5) from the same dogs were also calculated using Spearman rank correlation (r). Significance was set as *p* < 0.05.

## 3. Results

### 3.1. Semen Collection

The study included 33 ejaculates collected from 14 different dogs. Semen collection was performed twice on ten dogs, thrice on three dogs, and four times on one dog. All the patients included in the study were collected at T0 (n = 14). Eight semen samples were collected at T0.5, while n = 11 samples were obtained at T1. Semen collections at T1 were performed with a median time interval of 4 days (1–36 days) after T0. Information about the semen collection intervals for each patient included in the study is reported in Table 1.

### 3.2. Semen Analysis

The median semen concentration of all collected samples was 180 × 10^6^ (range 32–508) spermatozoa/ml. For semen morphology, a median value of 80% (range: 30–89%) normal spermatozoa, 6% (range: 0–18%) abnormal head, 2% (range: 0–6%) acrosomal abnormalities, 6.5% (range: 1–35%) abnormal tails, 2% (range: 0–26%) intermediate piece defects, 3% (range: 0–33%) detached heads, and 0% (range: 0–2%) presence of cytoplasmic droplets was reported. The descriptive data concerning mean and SD values of sperm morphology, total and progressive motility, and mean ± SD of proAKAP4 values are reported in Table 1. According to the proAKAP4 concentration interval classification, 17 semen samples (51%) were included in class 1 (≤15 ng/10 M spermatozoa), 8 samples (24%) in class 2 (15–40 ng/10 M spermatozoa), 2 samples (6%) in class 3 (40.01–60 ng/10 M spermatozoa), and 6 samples (18%) in class 4 (>60 ng/10 M spermatozoa).

### 3.3. ProAKAP4 Analysis

A slight correlation was found between proAKAP4 classes and TM% (r = 0.40, *p* = 0.049). Furthermore, no correlation was found between the proAKAP4 results and any sperm morphology parameter (Table 2).

Overall, no statistical differences were found for proAKAP4 classes between collection times (T0 vs. T0.5, *p* = 0.655; and T0 vs. T1, *p* = 0.564) (Table 3). No statistical differences concerning TM%, PM%, and concentration of spermatozoa were found at T0 vs. T0.5 (n = 8) (*p* = 0.07, *p* = 0.727, *p* = 0.727, respectively), as well as T0 vs. T1 (n = 9) (*p* = 0.82, *p* = 0.18, *p* = 0.51).

Concerning samples collected within the same day and in the same dog (T0 and T0.5), a statistically significant correlation was found between TM% and proAKAP4 classes (r = 0.66, *p* = 0.01) as well as between semen concentration and proAKAP4 classes (r = 0.70, *p* = 0.003). In contrast, no correlation was found between PM% and proAKAP4 classes (r = 0.014, *p* = 0.964).

## 4. Discussion

The research about proAKAP4 concentration and its relationship with other parameters commonly used for semen analysis is still lacking in dogs. Only two articles [29,39] and three abstracts [36,38,41] have been published. To the authors’ knowledge, this is the first study analyzing proAKAP4 concentrations in different semen ejaculates collected from the same dog at various time points.

Our study found an overall correlation between proAKAP4 classes and total motility. This result confirms what was reported in the previous literature in different species [6,7,31,33,35,42]. In the canine species, a positive correlation between proAKAP4 concentration and TM%, PM%, and velocity parameters (VAP, VSL, and VCL) has already been reported [36]. In bulls, proAKAP4 concentration is correlated to total and progressive motility, with higher proAKAP4 values in semen samples showing higher motility. ProAKAP4 has also been reported to be correlated with the linear and straightness motion of spermatozoa as well as with the beat cross frequency [27]. On the other hand, the influence of proAKAP4 values on velocity parameters (like VAP, VSL, and VCL) is still controversial [27,31,35,42]. In stallions, proAKAP4 is positively correlated with the total and progressive motility of frozen semen up to three hours after thawing [7], and it is also reported to be positively correlated with velocity parameters in thawed semen [33]. In cats, no correlations between proAKAP4 concentration and TM%, PM%, and velocity parameters were found [43].

Our study found no correlation between proAKAP4 classes and sperm morphological parameters. Specifically, no correlation with the percentage of abnormal tails was found. The statistical test may have failed to find significance because too-few samples had a significant percentage of abnormal tails. The relationship between proAKAP4 concentration and semen morphology is controversial. In bovines, De Almeida et al., 2022 [31], reported no relationship between proAKAP4 concentrations and the percentage of the observed morphological defects, mitochondrial functionality, and sperm membrane integrity. However, a previous study reported a positive correlation between the rate of normal spermatozoa and a negative correlation between the percentage of tail abnormalities and proAKAP4 values in frozen–thawed bull semen [35]. In human medicine, *AKAP4* gene alterations are reported in the morphological abnormalities of sperm tail [20,44,45]. Furthermore, studies on mice models reported a relationship between induced defects in the *AKAP4* gene and sperm flagellum alterations, resulting in immotile spermatozoa and mice infertility [20,21,22]. The relationship between proAKAP4 and morphological parameters should be re-evaluated in the future, especially considering the abnormalities of the tail and midpiece, as the activity of mitochondria enclosed in the midpiece is strongly connected to the total and progressive motility of the sperm cell.

In our study, no statistically significant difference was found between proAKAP4 classes at the first semen collection and the second semen collection when performed 2–3 h apart. The same result was observed for those performed between days 0 and 36 after the first semen collection. By performing this statistical evaluation, we aimed to verify the stability of proAKAP4 in semen samples collected twice within a short interval, to determine whether it could be considered similar to samples collected over a longer time interval. We decided to apply these two time intervals because motility and concentration parameters may change when two collections are performed within a short period. Specifically concentration, which seems to affect ProAKAP4, needs to be considered because it can interfere with the correct evaluation of proAKAP4.

Our results agree with the previous literature, where proAKAP4 concentration variability was studied in Landrace boars that underwent semen collection twice a week (day 0 and day 3) for 15 weeks [6,46]. They concluded that proAKAP4 concentration in boars seems to be stable in the same spermatogenetic cycle [6,46]. On the other hand, different proAKAP4 values were found in semen samples showing different concentrations in cats [43]. Few reports on modifying semen quality through repetitive semen collections are available in the canine species. Low sperm concentration was reported in ejaculates collected with a mean interval of 63 min from the same dogs [47]. However, no significant differences in total motility and morphology were reported between the first and second collected ejaculates [47]. Gunay et al. 2003 [48] evaluated the effect of two collected semen samples within 60 min in a group of adult, fertile, German shepherd stud dogs, stating that the semen quality and biochemical findings did not show any difference between the first and the second ejaculate, with comparable results for percentage of motility and live spermatozoa, acrosomal and tail abnormality, and total morphological defects, as well as plasma total protein, calcium, phosphorus, magnesium, sodium, and potassium [48].

In our study, no statistical difference was found in proAKAP4 between the ejaculates of semen samples belonging to the same dog collected on the same day. Also, a significant correlation was found between TM% and proAKAP4 classes (r = 0.66, *p* = 0.01). However, a significant correlation was found between semen concentration and proAKAP4 classes (r = 0.70, *p* = 0.003). With these evaluations, we tried to deepen the meaning of our results, keeping our attention focused only on ejaculates collected within the same dog in the time intervals considered. This also explains the higher level of significance of this analysis’ correlations with respect to the first one performed in all the samples. Vagenknechtova et al., 2010 [49], observed a reduction in sperm motility in ejaculates obtained from the same stud collected three times in the same week, ranging from 73 to 66% from the first to the third semen collection. However, no statistically significant difference was observed.

Furthermore, semen concentrations did not show any significant difference among ejaculates collected from the same dogs every 2 days for three times [49]. In a more recent study, Salvado et al. (2024) [50] studied the effect of five repetitions within two specific pre-established intervals (24 h vs. 48 h apart) in the Bull Terrier breed. They found that total sperm output and total sperm motility, vitality, and vigor decreased when the collection repetition was scheduled 24 h apart, meaning that too-intensive and too-frequent sessions of semen collection could impair semen quality. However, this happened just when the 24 h interval frequency of collection was maintained five times. On the other hand, when a 48 h interval between semen collection sessions was established, no alterations in semen quality were detected [50]. Sperm collection is usually not recommended to be repeated in a too-short interval of time on the same dog, because several parameters may be affected much more by the timing of collection than by the libido and current fertility of the stud dog, making it the interpretation of semen results complex and not conclusive. Despite this, in our experimental setting, semen parameters did not show statistically relevant alterations, and at a molecular level, proAKAP4 concentration was not modulated by serial sperm collections. Furthermore, it was considerably correlated to motility, as expected.

In the present report, proAKAP4 results were divided into four classes according to what was reported in the previous literature [6,46]. In a recent review, according to the results obtained by Sergeant et al., 2020 [46], on proAKAP4 concentration in boar ejaculates, four intervals of proAKAP4 concentration were established [6]. According to this classification, predicting long-lasting motility after 5 days of storage at 17 °C was possible, showing no differences in the pre-storage analysis (TM% and microscopic examination). The results obtained in our work about the correlation between proAKAP4 classes and motility agree with those previously reported, with this work being new for the canine species.

ProAKAP4 is a precursor polypeptide, and it must be converted into its active form (AKAP4) to impact sperm motility. Therefore, proAKAP4 concentration should be considered as a stock of motility of the examined semen sample, indicating the ability of spermatozoa to express its motility reserve and lifespan [5,7]. ProAKAP4 values could infer a poor motility reserve in fresh semen samples having suitable motility parameters. These characteristics make the proAKAP4 parameter a promising tool as a predictive marker of the future motility of the spermatozoa in the examined semen sample [6]. Further studies should be performed to analyze proAKAP4 values at different time points during the incubation of fresh semen samples in combination with other semen parameters to verify the predictive value of proAKAP4 in chilled and frozen–thawed semen motility parameters.

## 5. Conclusions

In conclusion, our study suggests that proAKAP4 levels do not to vary across different ejaculates collected at different times from the same dogs within the same spermatogenetic cycle. ProAKAP4 was distributed into four classes for comparison with sperm parameters. When considering samples collected from the same dog on the same day, a correlation was found between proAKAP4 classes and total motility and sperm concentration, suggesting that proAKAP4 concentration may be associated with sperm concentration in the canine species. Special attention should be paid to the time interval between collections when repetition is necessary. Additional information on this point could be obtained by altering the pattern of the semen collection, for example, by collecting semen within a week instead of on the same day. This would better determine the impact of a too-close semen collection that could impair seminal quality parameters, including proAKAP4. Moreover, further multicentric studies on a larger sample size are needed to (i) increase the number of patients having significant alterations in semen morphology, thus allowing a better focus on the correlations between morphology and proAKAP4 results; (ii) verify the proAKAP4 results in different samples representing different spermatogenetic cycles; and (iii) assess proAKAP4 results in samples under incubation for many hours after collection.

## Figures and Tables

**Table 1:** Mean ± SD of the main parameters considered in this study according to the collection times (T0: first semen collection; T0.5: semen collection performed between 2 and 3 h after T0; T1: semen collection performed between one and 35 days after T0.

	TM%	PM%	Norm %	Head Abn %	Acrosome %	Tail %	IP %	DH %	CD %
T0	90 ± 8.9	74.5 ± 12.2	78.3 ± 9.7	5.1 ± 2.2	1.6 ± 1.8	7.8 ± 5.4	4.1 ± 6.5	2.6 ± 2.5	0.5 ± 0.8
T0.5	91.9 ± 5.6	76.7 ± 11.1	74.5 ± 12.7	7.8 ± 4.6	3.1 ± 1.6	6.5 ± 3.5	2.6 ± 1.3	5.3 ± 5.8	0.3 ± 0.5
T1	86.8 ± 17.4	73.4 ± 19.3	76.7 ± 16.3	5.4 ± 2.5	1.2 ± 1.2	8.5 ± 9.3	1.9 ± 1.7	6.3 ± 9.3	0.1 ± 0.3

TM%: total motility; PM%: progressive motility; Norm%: normal spermatozoa; Head Abn%: abnormal head; Acrosome%: acrosome defects; Tail%: tail defects; IP%: intermediate piece defects; DH%: detached heads; CD%: cytoplasmatic droplets.

**Table 2:** Spearman rank correlation (r) and *p*-values calculated between proAKAP4 and morphology. Normal spermatozoa (spz), abnormal head (head), acrosomal abnormalities (acrosome), abnormal tails (tail), intermediate piece defects (IP), detached heads (DH), and presence of cytoplasmatic droplets (CD) were calculated on 100 examined spermatozoa after Spermac^®^ staining (Minitube, Tiefenbach, Germany) and reported in percentages (%). Significance was set as *p* < 0.05.

Variables	r	*p*-Value
Normal spz %	0.133	0.457
Head %	0.010	0.955
Acrosome %	0.130	0.469
Tail %	0.041	0.822
IP %	−0.326	0.065
DH %	−0.263	0.139
CD %	0.174	0.331

**Table 3:** Wilcoxon paired statistical test results of ProAKAP4 class distribution for the time intervals of semen collection considered. Results are presented as median and interquartile ranges. ProAKAP4 values are divided into four classes: class 1 = ≤15 ng/10 M spermatozoa, class 2 = 15–40 ng/10 M spermatozoa, class 3 = 40.01–60 ng/10 M spermatozoa, and class 4 = >60 ng/10 M spermatozoa [6]. Time 0 (T0): first semen collection performed on each dog included in the study, T0.5: semen collection performed between 2 and 3 h after T0, T1: semen collection performed between one and 35 days after T0.

	Time			*p*		
	0	0.5	1	0 vs. 0.5	0 vs. 1	0.5 vs. 1
n	14	8	11	8	9	3
cl ProAKAP4	2 (1–2.75)	1 (1–2.5)	1 (1–2)	0.655	0.564	-

## Data Availability

The authors have made the raw data supporting this article’s conclusions available in Appendix A and Appendix B.

## Appendix A

**Table A1:** Dog number, breed, date (day/month), day of semen collection (counted from the first ejaculate collected), and time class (first semen collection: T0, sampling performed between 2 and 3 h after T0: T0.5 and after one day or more: T1). Normal spermatozoa (spz), abnormal head (head), acrosomal abnormalities (acrosome), abnormal tails (tail), intermediate piece defects (IP), detached heads (DH), and presence of cytoplasmatic droplets (CD) were calculated on 100 examined spermatozoa after Spermac^®^ staining (Minitube, Germany) and reported in percentages (%) for each ejaculate.

Dog n.	Breed	Day of Semen Collection	Date (Year 2022)	Time Class	Normal Spz %	Head %	Acrosome %	Tail %	IP %	DH %	CD %
1	Great Dane	0	26/01	T0	88	5	0	3	2	2	0
		1	27/01	T1	74	6	2	7	2	9	0
		1	27/01	T1	82	4	0	2	5	6	1
2	Staffordshire Bull Terrier	0	03/02	T0	86	0	6	2	4	2	0
		7	10/02	T1	86	7	2	2	3	0	0
3	Mastiff	0	09/02	T0	81	7	1	8	1	2	0
		1	10/02	T1	79	6	2	9	1	3	0
4	Belgian Malinois	0	09/02	T0	75	6	0	12	4	3	0
		0	09/02	T0.5	68	9	3	9	3	8	0
5	Golden Retriever	0	07/02	T0	73	4	0	21	0	2	0
		1	08/02	T1	80	8	3	8	1	0	0
6	Newfoundland	0	07/02	T0	52	9	0	12	26	1	0
		0	07/02	T0.5	45	18	5	13	1	18	0
		9	16/02	T1	30	1	0	35	1	33	0
		14	22/02	T1	81	6	0	7	1	5	0
7	Pug	0	08/02	T0	73	4	4	8	5	4	2
		5	13/02	T1	72	3	2	11	5	7	0
8	Musterlander	0	14/02	T0	88	6	1	1	4	0	0
		0	14/02	T0.5	80	8	5	4	3	0	0
9	American Staffordshire Terrier	0	14/02	T0	82	7	3	7	1	0	0
		2	16/02	T1	87	3	2	4	1	3	0
10	Border Collie	0	16/02	T0	86	5	2	4	1	0	2
		0	16/02	T0.5	81	8	1	5	2	2	1
11	Jack Russell	0	22/02	T0	80	2	2	10	2	4	0
		0	22/02	T0.5	80	5	4	4	1	6	0
		36	30/03	T1	84	10	0	2	1	3	0
12	Jack Russell	0	28/02	T0	75	6	1	5	4	8	1
		0	28/02	T0.5	81	5	3	3	3	5	0
13	Newfoundland	0	01/03	T0	86	5	0	4	3	1	1
		0	01/03	T0.5	80	6	3	5	3	2	1
14	Welsh Corgi Pembroke	0	28/03	T0	71	5	3	12	1	7	1
		0	28/03	T0.5	81	3	1	9	5	1	0
		3	31/03	T1	89	5	0	6	0	0	0

## Appendix B

**Table A2:** Dog number, breed, and time class (first semen collection: T0, semen collection performed between 2 and 3 h after T0: T0.5 and after one day or more: T1). Total (TM%) and progressive (PM%) motility are measured using a Computer-Assisted Sperm Analyser (CASA, Ceros II, Hamilton Thorne Inc., USA) and reported in percentages. ProAKAP4 values are reported as nanograms on 10 million spermatozoa (ng/10 M spermatozoa) and four intervals defined as class 1: ≤15, class 2: 15–40, class 3: 40.01–60, and class 4: >60 ng/10 M spermatozoa [6,32].

Dog n.	Breed	Time Class	TM %	PM %	ProAKAP4 Class	ProAKAP4 (ng/10 M Spermatozoa)
1	Great Dane	T0	93.6	78	2	31.05
		T1	93.4	82.1	2	20.15
		T1	94.7	86.4	1	14.55
2	Staffordshire Bull Terrier	T0	94.1	78.4	1	6.95
		T1	94.5	73.1	1	4.63
3	Mastiff	T0	93.4	70	2	21.13
		T1	91.5	76.3	2	25.71
4	Belgian Malinois	T0	89.4	79.2	2	16.37
		T0.5	94.9	78.2	2	20.12
5	Golden Retriever	T0	94.7	82.4	4	78.9
		T1	91.2	85.7	3	47.02
6	Newfoundland	T0	61.8	37.1	1	3.59
		T0.5	84.3	65.4	1	10.86
		T1	34.8	17.9	1	10.2
		T1	93.3	78.4	1	13.37
7	Pug	T0	94.3	73.5	2	23.74
		T1	91.3	81.7	1	5.33
8	Musterlander	T0	91.3	81.2	1	11.02
		T0.5	94.7	79.7	4	176.72
9	American Staffordshire Terrier	T0	88.7	80.3	4	77.09
		T1	92.1	82.2	4	62.27
10	Border Collie	T0	94.7	80	3	53.15
		T0.5	95.1	87	1	5.46
11	Jack Russell	T0	89.4	67	1	7.61
		T0.5	91.8	87.8	1	9.8
		T1	87.2	77.2	1	3.91
12	Jack Russell	T0	93.4	87.7	1	9.67
		T0.5	93.5	84.2	1	14.23
13	Newfoundland	T0	98.2	80.3	4	101.58
		T0.5	98.4	75.5	4	65.44
14	Welsh Corgi Pembroke	T0	83.1	68.2	1	11.35
		T0.5	82.4	55.8	1	4.28
		T1	91	66.2	2	15.87
